# Cholesterol Microlithiasis: Bacteriology, Gallbladder Bile and Stone Composition

**DOI:** 10.1155/1989/80465

**Published:** 1989

**Authors:** Leopoldo Sarli, Matteo Gafa', Ernesto Longinotti, Fabio Carreras, Nicola Pietra, Anacleto Peracchia, Claudio Dotti, Simonetta Cavalier

**Affiliations:** 1 Institute of “Clinica Chirurgica Generale II e Terapia Chirurgica” Parma University Italy; 2 Department of Radiology “I Servizio, Sezione Radioisotopi” of “S. Maria Nuova Hospital” Reggio Emilia Italy; 3 c/o Clinica Chirurgica II dell ‘Universita’ Ospedale Maggiore Via Gramsci n. 14 Parma 43100 Italy

## Abstract

It is not known whether microcalculi possess structural differences compared with larger stones or whether they represent simply an earlier stage in stone disease.

We carried out a controlled study on 10 patients affected by gallbladder cholesterol microlithiasis (CM). In all patients, samples from all parts of the stones were studied by X-ray diffraction and by infrared spectrophotometry. Bile analysis was carried out to determine cholesterol, phospholipid and total bile acid content. The cholesterol saturation indices (C.S.I.) were calculated. In all samples, bacterial bile culture was carried out. The results were compared with those of 10 patients who had undergone cholecystectomy for large cholesterol stones, and for 10 patients who had undergone abdominal surgery but without biliary pathology. Patients in these latter groups were matched with the first according to sex and age.

Microcalculi proved to be layered (nucleus and external layer) in only 2 cases and larger stones in 9; cholesterol was seen to be the principal crystalline component in all cases. Traces of bilirubin were found in 7 CM and in the nuclei of 5 larger stones.

These results show that the structural composition of microcalculi is similar to that of the nucleus of larger stones. No substantial differences exist, however, between the two groups of patients regarding the other parameters taken into consideration.


*HPB Surgery* 1989, Vol. 1, pp. 283–295
Reprints available directly from the publisher
Photocopying permitted by license only

© 1989 Harwood Academic Publishers GmbH
Printed in Great Britain

### CHOLESTEROL MICROLITHIASIS: BACTERIOLOGY, GALLBLADDER BILE AND STONE COMPOSITION

LEOPOLDO SARLI*, MATTEO GAFA', ERNESTO LONGINOTTI, FABIO CARRERAS, NICOLA PIETRA and ANACLETO PERACCHIA

*Institute of "Clinica Chirurgica Generale II e Terapia Chirurgica" of Parma University, Italy*

CLAUDIO DOTTI and SIMONETTA CAVALIER

*Department of Radiology "I Servizio, Sezione Radioisotopi" of "S. Maria Nuova Hospital" Reggio Emilia, Italy*

*(Received 17 March 1988)*

It is not known whether microcalculi possess structural differences compared with larger stones or whether they represent simply an earlier stage in stone disease.

We carried out a controlled study on 10 patients affected by gallbladder cholesterol microlithiasis (CM). In all patients, samples from all parts of the stones were studied by X-ray diffraction and by infrared spectrophotometry. Bile analysis was carried out to determine cholesterol, phospholipid and total bile acid content. The cholesterol saturation indices (C.S.I.) were calculated. In all samples, bacterial bile culture was carried out. The results were compared with those of 10 patients who had undergone cholecystectomy for large cholesterol stones, and for 10 patients who had undergone abdominal surgery but without biliary pathology. Patients in these latter groups were matched with the first according to sex and age.

Microcalculi proved to be layered (nucleus and external layer) in only 2 cases and larger stones in 9; cholesterol was seen to be the principal crystalline component in all cases. Traces of bilirubin were found in 7 CM and in the nuclei of 5 larger stones.

These results show that the structural composition of microcalculi is similar to that of the nucleus of larger stones. No substantial differences exist, however, between the two groups of patients regarding the other parameters taken into consideration.

KEY WORDS: Gallstone composition, bile composition, bile bacteria, gallbladder physiopathology, biliary tract disease.

### INTRODUCTION

Biliary microlithiasis is defined as gallstones less than 3 mm. in diameter[1,2]. In earlier clinical studies,[3,4,5] biliary microcalculi have been observed to have particular characteristics absent in larger stones. Colic pain caused by "migration" and acute obstruction of the papilla of Vater, may contribute to the high incidence of acute pancreatitis, associated with this disease. It is not yet clear whether, as seems reasonable to suppose, microcalculi are the first stage in larger stone formation or if they possess structural peculiarities which impede their growth.

We therefore performed a controlled study on patients affected by biliary microlithiasis to examine the chemical structure of the concretions, bile composition and bile bacteriology.

Author responsible for correspondence regarding the manuscript and to whom request reprints should be addressed: Dr. Leopoldo Sarli*, c/o Clinica Chirurgica II dell 'Universita', Ospedale Maggiore, Via Gramsci n. 14, 43100 Parma, Italy

### MATERIALS AND METHODS

The study was carried out on 30 patients divided into 3 groups. 10 patients (group 1) were affected by gallbladder microlithiasis; the microliths, of homogeneous dimensions, classified by visual inspection as cholesterol type, were removed at consecutive operations between March 1985 and March 1987. 10 patients (group II), who underwent elective cholecystectomy for larger gallstones in the same period, were matched with group I according to sex and age (plus or minus 4 years); the stones of homogeneous dimensions, were morphologically classified as cholesterol type. 10 patients (group III), matched with the other groups according to sex and age, underwent non-biliary abdominal surgery. This group was without biliary tract disease, as assessed by preoperative ultrasonography and intraoperative evaluation.

None of the patients in group I and II had common duct lithiasis as assessed by intraoperative cholangiography; none was obese or diabetic. Preoperative liver function tests were normal in all cases. None received preoperative antibiotics.

Each group of patients comprised 4 males and 6 females. The mean age was 52.2 ± 13.1 years ranging from 29 to 71 years in group I; 51.5 ± 13.2 ranging from 27 to 70 years in group II and 51.8 ± 13.2 ranging from 32 to 72 years in group III.

In all patients gallbladder bile samples were obtained anaerobically at surgery by needle puncture aspiration. Aspiration of gallbladder content was complete in order to reduce, as far as possible, sampling errors due to bile stratification[6]. In groups I and II at least one stone was taken in each case; the stones obtained were washed several times with distilled water and air-dried; they were then cross-sectioned and examined microscopically in order to reveal the possible presence of concentric or superimposed layers.

### *Analysis of Stone Composition*

Gallstones collected from excised gallbladders were investigated immediately after operation (usually on the same day). All samples, consisting of untreated substance from all parts of the calculi, were studied by X-ray diffraction and by infrared spectrophotometry.

Since, in case of microlithiasis, we did not have a sufficient quantity of substance for common diffractometry and recording on paper, all the stones were analyzed using the single rotating crystal (Debye-Scherrer method) with Gandolfi camera and recorded on photographic film.

A small fragment, of micron order, of each layer was used for X-ray diffraction analysis (Cu-KBr tube), with exposure times of 4 hours. Reading and interpretation was helped by the A.S.T.M.* international sheets[6,7]. The quantity of amorphous substance was not determined.

Infrared spectra were obtained using the PYE-UNICAM SP-3 200S I.R. spectrophotometer with KBr pellet technique. The remaining part of the stone was pulverized in a mortar. The components of the stones were identified by comparison with pure standards (cholesterol and bilirubin) or by using data published in the literature (palmitate and carbonate). To determine the quantities of cholesterol and bilirubin, mixtures of pure standards were prepared in KBr with concentrations varying from 0.10 to 0.80% weight/weight. To obtain a uniform thickness in all

*A.S.T.M. American Standards of Testing Minerals samples, the preparation of the pellet was standardized in the following way: 0.6–0.7 mg. of the stone powder were mixed in a mortar for 5 minutes with 120–130 mg. of KBr; from this mixture two pellets of equal weight were obtained using a hydraulic press (12 lb./sq. in. for 2 minutes). The following signficance frequencies were used: bilirubin = 1620 $cm^{-1}$, cholesterol = 2940 $cm^{-1}$. For cholesterol this frequency was used because comparative tests revealed it to be less sensitive to interference from bilirubin than the frequency of 3600 $cm^{-1}$ commonly used. The absorbance of each peak was calculated using the base-line method. By plotting the concentration values against the absorbance of the standards, a linear regression was obtained with the following coefficients: cholesterol $x = 0.001 + 0.663\ y$ and $r^2 = 0.990$; bilirubin $x = 0.019 + 0.841\ y$ and $r^2 = 0.991$. By placing on calibration graphs the absorbance of the stones, the concentration of the various components present in each stone was obtained[8,9]. Other amorphous components were not evaluated by spectrophotometry.

### *Analysis of Bile Composition*

Bile was kept at −20°C for 5–7 days. After thawing, it was used for the determination of cholesterol content (Boehringer-Mannheim enzymatic-colorimetric method), phospholipid content (Boehringer- Mannheim molybdate-vanadate reaction by precipitation), and total biliary acid content (Sterognost 3 alpha Nyegaard).

A saturation index which take into account the total lipid concentration was calculated according to Carey[12].

### *Bacteriology*

Undiluted samples of gallbladder bile for culture studies were obtained by a sterile technique and immediately placed in prereduced anaerobically sterilized transport tubes. The material was used to detect aerobic and anaerobic micro-organisms. Anaerobic bacteria were identified by gas-chromatography, aerobic bacteria by conventional methods. Bacterial counts was obtained with scalar dilutions of the test sample. Counts higher than 100000/ml were considered positive.

### *Statistical Evaluation*

Student's T test was used to evaluate the significance of differences in mean values.

### RESULTS

### *Bile Composition*

The total and relative composition of the bile samples from microcalculi patients, large-stone patients and control non-gallstone patients is given in Table 1.

The results show a significant difference ($p < 0.05$) between microcalculi and control patients in percentage of cholesterol present in the bile and in saturation index calculated by all three methods. No significant differences were revealed, however, between microcalculi and larger stone patients and between larger stone patients and control patients. It is noteworthy that in microcalculi patients bile is always revealed to be supersaturated, whereas the bile of large-stone patients is not supersaturated only in 3 cases.

**Table 1** Total and relative biliary lipid composition data, calculated cholesterol (ch) saturation indices (C.S.I.)* and microbiological findings.

| | *Composition (% total mol.)* | | | *Total lipids* | | *Positive* |
|---|---|---|---|---|---|---|
| | *Bile acids* | *Lecithin* | *Ch* | *g/dl* | *C.S.I.* | *bile colture* |
| | Value of gallbladder biles from cholesterol gallstone patients | | | | | |
| Mean | 72.19 | 18.54 | 9.27 | 9.39 | 1.58 | 10% |
| ±SD | 3.92 | 4.62 | 2.33 | 3.61 | 0.61 | |
| | Values of gallbladder biles from cholesterol microcalculi patients | | | | | |
| Mean | 67.19 | 21.4°° | 11.39° | 10.90 | 1.60° | 20% |
| ±SD | 4.78 | 3.79 | 2.54 | 4.24 | 0.36 | |
| | Values of gallbladder biles from control non-gallstone patients | | | | | |
| Mean | 72.04 | 20.08 | 7.88 | 9.89 | 1.18 | 20% |
| ±SD | 4.95 | 4.21 | 2.79 | 4.89 | 0.43 | |

* C.S.I. have been calculated using the limits of cholesterol solubility as defined by Carey (14).
°° Significantly different ($p < 0.025$) from the corresponding index in control patients.
° Significantly different ($p < 0.05$) from the corresponding index in control patients.

### *Stone Composition* (Tables 2 and 3)

Under the optical microscope, the microcalculi were shown to be without layers (Figure 1) with the exception of two cases in which it was possible to distinguish a nucleus and an outer layer. In 9 cases (90%), cholesterol proved to be the only crystalline component on diffractometry (Table 2). In 2 cases in which a nucleus and an outer layer were present, cholesterol proved to be the only crystalline component in both layers. In one case (10%), together with the cholesterol, calcium carbonate was present in the form of vaterite. A ring corresponding to amorphous substance was present in 9 cases in the Debye reading, but the diffuse in only one case. In those having a nucleus and an outer layer, amorphous substances were found only in the nucleus. Large stones were shown to be layered in 9 cases (Figure 1). Cholesterol was also present as a crystalline component (Table 3) in all the cases, both in the nucleus and in the outer layers. Calcium carbonate in the form of vaterite was found in 5 cases: in 4 in the outer layer and in 1 in both the nucleus and the outer layer; in the form of calcite it was observed in the external covering of 1 stone only (10%). Debye reading revealed a ring corresponding to amorphous substances in 5 cases: in 2 cases both in the nucleus and in the external layer, in 3 cases only in the nucleus.

Infrared spectrophotometry (Tables 2 and 3) provided further details concerning the substance which had appeared amorphous at diffractometry, revealing the presence of bilirubin in 7 cases of microcalculi and in 5 of large stones where the Debye test showed rings of amorphous substance. No correlations were observed between bile composition and stone composition for any of the parameters examined.

### *Bile Bacteriology* (Tables 1, 2 and 3)

Bile bacteria were found in 2 microcalculi patients (20%), in 1 large-stone patient (10%) and in 2 non-gallstone control patients (20%). No correlations were observed between the presence of bacteria in the bile and the composition of stones. A very low concentration of total lipids was found, however, in the bile in cases of bile bacteria.

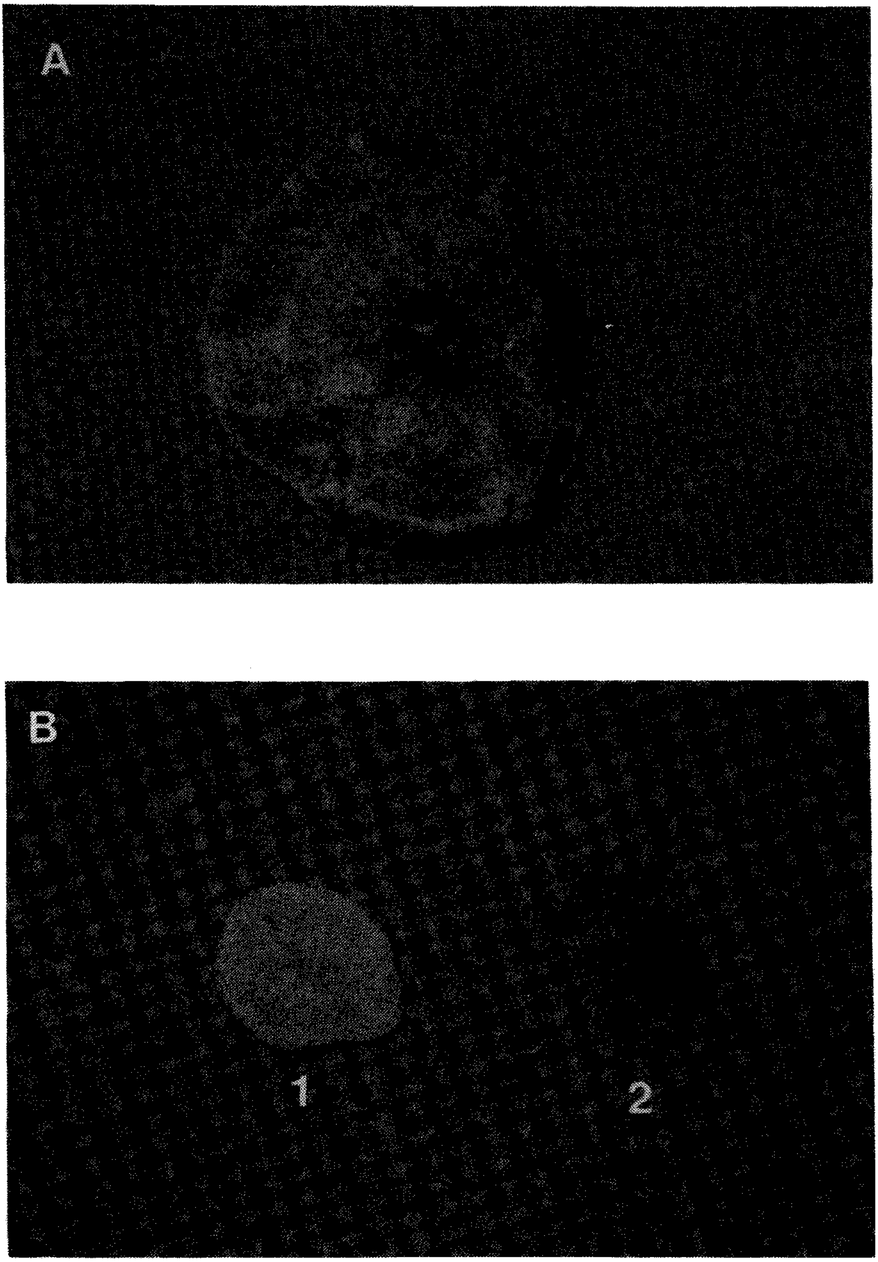

**Figure 1** A) Stratified larger stone with a nucleus containing bilirubin. (× 10) B) Cholesterol microcalculi not stratified. The microlith number 2 contains bilirubin like the nucleus of larger stone. (× 25)

**Table 2** Components of cholesterol microcalculi as defined by spectroscopy and diffractometry.

| | *Microscopic features* | *Composition* | *%* | *Bacteriological bile colture* |
|---|---|---|---|---|
| 1. | coating | cholesterol | 95 | negative |
| | nucleus | cholesterol | 60 | |
| | | bilirubin | traces | |
| | | amorphous subst. | n.c. | |
| 2. | homogeneous | cholesterol | 90 | negative |
| | | bilirubin | 3 | negative |
| | | amorphous subst. | n.c. | |
| 3. | homogeneous | cholesterol | 95 | positive |
| 4. | homogeneous | cholesterol | 85 | negative |
| | | bilirubin | traces | |
| | | amorphous subst. | n.c. | |
| 5. | homogeneous | cholesterol | 90 | negative |
| | | amorphous subst. | n.c. | |
| 6. | homogeneous | cholesterol | 85 | negative |
| | | amorphous subst. | n.c. | |
| 7. | homogeneous | cholesterol | 80 | positive |
| | | bilirubin | 6 | |
| | | amorphous subst. | n.c. | |
| 8. | homogeneous | cholesterol | 70 | negative |
| | | vaterite | 10 | |
| | | bilirubin | traces | |
| | | amorphous subst. | n.c. | |
| 9. | homogeneous | cholesterol | 85 | negative |
| | | bilirubin | traces | |
| | | amorphous subst. | n.c. | |
| 10. | coating | cholesterol | 88 | negative |
| | | bilirubin | 5 | |
| | | amorphous subst. | n.c. | |
| | nucleus | colesterol | 60 | |
| | | bilirubin | 5 | |
| | | amorphous subst. | n.c. | |

n.c. = not calculated

Our examinations enabled us to single out 100% of stone components. A small quantity of both coating and nucleus components remained undetected.

### DISCUSSION

The following significant data emerge from our study: microcalculi rarely present layers, whereas larger stones always have a nucleus and an outer layer; the composition of microcalculi is similar to that of the nucleus of larger stones, the calcium carbonate often present in the outer layers of larger stones, being rarely present in their nuclei or in microcalculi; furthermore, traces of bilirubin are rarely detectable in the outer layers of larger stones, while they are often found in their nuclei or in microcalculi. No substantial differences were noted between the two groups of patients for the parameters we considered as to gallbladder bile composition and bile bacteriology. These data would seem to lend weight to the hypothesis of microcalculi as being "young stones" and representing an early stage in

**Table 3** Components of cholesterol larger stones as defined by spectroscopy and diffractometry.

| *Stone size* | *Microscopic features* | *Composition* | % | *Bacteriological bile colture* |
|---|---|---|---|---|
| 1. medium | coating | cholesterol | 88 | negative |
| | | vaterite | 10 | |
| | nucleus | cholesterol | 88 | |
| | | bilirubin | 7 | |
| | | amorph. subst. | n.c. | |
| 2. medium | coating | cholesterol | 80 | negative |
| | | vaterite | 17 | |
| | nucleus | cholesterol | 70 | |
| | | bilirubin | 5 | |
| | | amorph. subst. | n.c. | |
| 3. large | coating | cholesterol | 75 | negative |
| | | calcite | 20 | |
| | nucleus | cholesterol | 95 | |
| 4. small | coating | cholesterol | 86 | positive |
| | | ca. palmitate | 3 | |
| | | bilirubin | 5 | |
| | nucleus | cholesterol | 90 | |
| | | amorph. subst. | n.c. | |
| 5. small | homogeneous | cholesterol | 80 | negative |
| | | amorph. subst. | n.c. | |
| 6. medium | coating | cholesterol | 75 | negative |
| | | vaterite | 23 | |
| | nucleus | cholesterol | 98 | |
| 7. large | coating | cholesterol | 75 | negative |
| | | vaterite | 20 | |
| | nucleus | cholesterol | 70 | |
| | | vaterite | 27 | |
| 8. medium | coating | cholesterol | 80 | negative |
| | | vaterite | 18 | |
| | nucleus | cholesterol | 95 | |
| 9. small | coating | cholesterol | 98 | negative |
| | nucleus | cholesterol | 80 | |
| | | bilirubin | 3 | |
| | | amorph. subst. | n.c. | |
| 10. small | coating | cholesterol | 85 | negative |
| | | bilirubin | traces | |
| | | amorph. subst. | n.c. | |
| | nucleus | cholesterol | 80 | |
| | | bilirubin | traces | |
| | | amorph. subst. | n.c. | |

n.c. = not calculated

Our examinations enabled us to single out 100% of stone components. A small quantity of both coating and nucleus components remained undetected.

the formation of a larger stone, often its nucleus.

This is supported by the results concerning gallbladder bile composition: all microcalculus patients had cholesterol-supersaturated bile while two large stone patients had non-supersaturated bile.

These observations suggest that gallbladder bile containing microcalculi still preserves the conditions which originally caused lithogenesis and thus microliths could be used to study calculus formation. In fact, although cholesterol supersaturation is considered a prerequisite to cholesterol gallstone formation, this is not the only casue since most normal subjects in the western world, as confirmed by our analysis, have a cholesterol-supersaturated bile. We came to the conclusion that there are many factors involved in the pathogenesis of calculi[13]; in recent studies particular stress has been laid on the nucleation of cholesterol crystals as an essential stage in gallstone formation[14,15]. These observations are based to a great extent on animal models of cholesterol lithiasis[16], from phase-diagrams obtained in vitro using model solution of cholesterol, bile acids and lecithin[12]; the data obtained are not always applicable to human bile[13]. One reason for the difficulty of researchers to identify the first stages and thus the exact pathogenesis of biliary concretions in man, is that calculi often show up years or even decades after their formation, when the conditions which caused lithogenesis are no longer present. That is why microliths, should they be confirmed as being of recent formation, could be useful in the study of lithogenesis. Our results, however are derived from too small a number of cases, and they must be confirmed. It could be very useful to carry out new comparative studies on large-stone patients and microcalculi patients, evaluating concentration and composition of apolipoproteins in the bile and the nucleation time which are very important with respect to lithogenesis[13].

Bacterial infection has been proposed as a possible cause of gallstone formation and bacteria have been suggested as nucleant factors in the crystallization of cholesterol[17].

In this study, as in those of other authors[18,19], infected bile showed an abnormally low total lipid content which may itself be a cause for bile lithogenicity[12]. Recent studies, however, suggest that bacterial infection is probably not important for cholesterol stone formation[20,21]. Our results support this second hypothesis; indeed, bile bacteria have been found in microcalculus patients, in large-stone patients, and in non-gallstone controls. In conclusion, cholesterol microcalculi do not seem to be structurally different from larger stone in that they are similar in their composition to the nuclei of larger stones[22]. It is possible that they represent an early stage in the natural history of the stone. More detailed analysis of the structure of microcalculi and bile that contains them could thus be useful in the search for the exact pathogenesis of biliary lithiasis.

### *References*

1. Houssin, D., Castaing, D., Lemoine, J. and Bismuth, H. (1983). Microlithiasis of the gallbladder. *Surgery Gynecology & Obstetrics,* **157**, 20–24.
2. Bertrand, L. and Lamarque, J.L. (1975) La microlithiase biliaire. *Nouvelle Presse Médical,* **4**, 3135–3138.
3. Peracchia, A., Lupi, M., Sarli, L., Gafa', M., (1984) Diagnosi e terapia della microlitiasi biliare. *Chirurgia Oggi,* **150**, 503–506.
4. Farinon, A.M., Ricci, G.L., Sianesi, M., Percudani, M. and Zanella, E. (1987) Physiopathological role of microlithiasis in gallstone pancreatitis. *Surgery Gynecology & Obstetrics,* **164**, 252–256.

5. Peracchia, A., Gafa', M., Sarli, L., Lupi, M. and Longinotti, E. (1985). Biliary microlithiasis and acute pancreatitis. *International Surgery,* **70**, 315–318
6. Tera, H. (1960) Stratification of human gallbladder bile vivo. *Acta Chirurgica Scandinavica Suppl.,* **256**, 1–85
7. Kapur, M.L. and Ananthakrishnan, N. (1982) Combined X-ray diffraction and microchemical gallstone analysis in determining the sequential events in biliary lithogenesis. *International Surgery,* **67**, 169–173
8. Bogren, H. and Larsson, K. (1963) Crystalline components of biliary calculi. *Scandinavian Journal of Clinical and Laboratory Investigation,* **15**, 557–558
9. Trotman, B.W., Morris, T.A., Sanchez, H.M., Soloway, L.D. and Ostrow, J.D. (1977) Pigment versus cholesterol cholelithiasis: identification and qualification by infrared spectroscopy. *Gastroenterology,* **72**, 495–504
10. Toyoda, M. (1966) Qualitative determination of calcium bilirubinate in gallstone by infrared spectroscopy. *Tohaku journal of Experimental Medicine,* **90**, 303–308
11. Trotman, B.W., Ostrow, J.D. and Soloway, R.D. (1976) Pigment versus cholesterol cholelithiasis: comparison of stone and bile composition. *American Journal of Digestive Diseases,* **7**, 19, 585–590.
12. Carey, M.C. and Small, D.M. (1978) The physical chemistry of cholesterol solubility in bile. Relationship in gallstone formation and dissolution in man. *Journal of Clinical Investigation,* **61**, 998–1026
13. Holan, K.R., Holzbach, R.T., Hermann, R.E., Cooperman, A.M. and Claffey, W.I. (1979) Nucleation time: a key factor in the pathogenesis of cholesterol gallstone disease. *Gastroenterology,* **77**, 611–617.
14. Smith, B.F., La Mont, J.T. and Small, D.M. (1987) The sequence of events in gallstone formation. *Laboratory Investigation,* **56**, 125–126
15. Hozbach, R.T. (1986) Recent progress in understanding cholesterol crystal nucleation as a precursor to human gallstone formation. *Hepatology,* **6**, 1403–1406
16. Mac Person, B.R., Pemsingh, R.S. and Scott, G.W. (1987) Experimental cholelithiasis in the ground squirrel. *Laboratory Investigation,* **56**, 138–41
17. Small, D.M. (1980) Cholesterol nucleation and growth in gallstone formation. *New England Journal of Medicine,* **302**, 1305–1311
18. Angelico, M., Attili, A.F., Cantafora, A., Bracci, F., Panichi, G., Pieche, U., Alvaro, D. and Capocaccia, L. (1982) Stone composition and gallbladder bile analysis and bacteriology in patients with radiolucent gallstones. Comparison with control subjects. *Italian Journal of Gastroenterology,* **14**, 139–144
19. Goodhart, G.L., Levison, M.E., Trotman, B.W. and Soloway, R.D. (1978) Pigment versus cholesterol cholelithiasis. Bacteriology of gallbladder stone, bile and tissue correlated with biliary lipid analysis. *American Journal of Digestive Diseases,* **23**, 877–882
20. Masuda, H. and Nakajama, F. (1979) Composition of bile pigment in gallstones and bile and their etiological significance. *Journal of Laboratory and Clinical Medicine,* **93**, 353–360
21. Chainoff, C.H. and Menache, R. (1979) Bile composition and osmolarity in the interpretation of formation of bile stone and their classification. *British Journal of Surgery,* **66**, 476–477
22. Gafa', M., Sarli, L., Bonilauri, E., Longinotti, E., Carreras, F., Pietra, N. and Peracchia, A. (1988) Composition of biliary microcalculi studied with infrared spectrophotometry. *Acta Chirurgica Scandinavica,* **154**, 195–198

### INVITED COMMENTARY

A peculiar feature of cholelithiasis is the fact that the size and number of stones present in the gallbladder can vary enormously. The world record for the number of gallstones found in a gallbladder has been discussed recently and is thought to be 36,329 stones[1].

While multiple, small stones are common, around 1 in 5 gallstone patients will have a solitary stone. The factors that control size and number are not understood.

In an Australian survey of 387 consecutive gallstone patients treated by cholecystectomy, 82% were found to have multiple stones in their gallbladder, as opposed to a solitary stone[2]. In addition, the total mass of gallbladder stones, which were high in cholesterol content ranged from 0.1 to 47.0 grams. In this group of stones, the total mass of stones and the diameter of the largest stone increased with the age of the patient (2). This finding supports the concept that stones grow slowly over a period of many years. Recent quantitative information from radiocarbon dating suggested that the growth rate is 2.6 mm increase in stone diameter per year[3].

The study by Sarli and his colleagues has compared the chemical composition of microcalculi (< 3 mm diameter) with that of larger stones, each stone type being collected from a group of 10 gallstone patients. Both X-ray diffraction and infra-red spectroscopy were used to detect crystalline components, such as cholesterol monohydrate, bilirubin and calcium salts. All stones were found to be predominantly composed of cholesterol, but layers were frequent in the larger stones, with calcium salts (vaterite, palmitate) present in the outer layer. This latter phenomenon has been described in studies of gallbladder stones which were resistant to bile acid dissolution therapy[4]. Overall, the microcalculi were very similar chemically to the nucleus of larger stones, a finding consistent with the notion that they simply represent a transition stage in stone development.

A high cholesterol saturation index for gallbladder bile is associated with cholesterol gallstone information, but nucleating factors also are required to induce cholesterol crystallization[5]. A reasonable hypothesis as to why some patients develop multiple small stones, while others form a single large stone, is that a 'shower' of micronuclei could provide multiple sites for stone growth, whereas a limited number of micronuclei could induce the formation of a few cholesterol crystals, which if aggregated would result in a solitary stone. The chemical nature of nucleation-promoting factors remains to be fully elucidated[6]. In the study of Sarli *et al.*, micro-biological examination of gallbladder bile did not provide evidence for bacteria as nucleating factor.

Whether stones continue to grow is probably controlled by the continued presence of bile which is supersaturated with cholesterol and stone surfacc characteristics favourable to further precipitation. Calcium salts appear to retard cholesterol deposition, as shown by stones with calcified rims. The degree of biliary cholesterol supersaturation also may be important in determining stone size and number. Obese patients undergoing weight reduction after gastric bypass surgery are thought to have a transient, very high bile cholesterol saturation. Many of these patients develop multiple microliths[7] of the new type analysed by Sarli *et al.* in their work.

### *References*

1. Nance, M.L. (1988). Record number of gallstones (letter) *Brit. J. Surg.*, **75**, 93
2. Whiting, M.J., Bradley, B.M. and Watts, J.McK. (1981). Chemical and physical properties of gallstones in South Australia. *Gut*, **24**, 11–15
3. Mok, H.Y., Druffel, C.R. and Rampone, W.M. (1986). Chronology of cholclithiasis. *New Eng. J. Med.*, **314**, 1075–1077
4. Whiting, M.J., Jarvinen, V. and Watts, J.McK (1980). Chemical composition of gallstones resistant to dissolution therapy with chenodeoxycholic acid. *Gut*, **21**, 1077–1081
5. Smith, B.F. and LaMont, J.T. (1986). The central issue of cholesterol gallstones. *Hepatology*, **6**, 529–531
6. Burnstein, M.J., Ilson, R.G., Petrunka, C.N., Taylor, R.D. and Strasberg, S.M. (1983). Evidence for a potent nucleating factor in the gallbladder bile of patients with cholesterol gallstones. *Gastroenterology*, **85**, 801–807

7. Wattchow, D.A., Hall, J.C., Whiting, M.J., Bradley, B.M., Iannos, J. and Watts, J.McK. (1983). Prevalence and treatment of gallstones after gastric bypass surgery for morbid obesity. *Brit. Med. J.*, **286**, 763

Malcolm Whiting
Department of Biochemistry and Chemical Pathology
Flinders Medical Centre
Bedford Park
Australia

### INVITED COMMENTARY

The presented paper by Sarli and coworkers deals with the importance of biliary microcalculi in the pathogenesis of larger gallstones. They postulate that, due to the chemical similarly between microlithiasis and the nuclei of larger concretions, microcalculi are "young stones", representing an early stage in the formation of larger conrements.

This raises the question of early detection of these stone precursors. Conventional roentgenologic techniques, such as plain abdominal X-ray and inatravenous cholangiography are not sensitive enough to detect microcalculi, which are, by definition, smaller than 3 millimeter in diameter[1]. Computed tomography, from our experiences does not allow the detection of pure cholesterol stones, probably due to the similar densities of fluid and crystalline cholesterol[2]. Therefore, only abdominal ultrasound serves as an adequate diagnostic tool in the detection of small gallbladder stones.

But, there are also some limitations of this technique. It is of upmost importance to use high frequency transducers (5 MHz and more) to obtain excellent resolution and thereby reproducible results. The resolution of a pulse-echo-system is determined by the wave length (λ) of the applied ultrasound frequency (f). Separation of different reflectors on the ultrasound screen can be achieved only in those cases where the reflector distance is greater than the pulse wave length. This theoretical resolution of an ultrasound system can be calculated by the formula[3]

$$\lambda = \frac{v}{f}$$

where the velocity (v) of the ultrasound beam in the human body is assumed to be 1500 m/sec. Therefore, the resolution of a 3 MHz transducer is

$$\lambda_3 = \frac{1500\ \text{m/sec}}{3\ \text{MHz}} = 0.5\ \text{mm}$$

In the case of a MHz transducer, as recommended above, the resolution improves to

$$\lambda_5 = \frac{1500\ \text{m/sec}}{5\ \text{MHz}} = 0.3\ \text{mm}$$

Unfortunately ultrasound pulses are not propagated as ideal sinus wave oscillations. The real resolution of an ultrasound system in the perpendicular axis, called acial resolution, is diminished by "after ringing" of the applied ceramic and therefore depends on the damping characteristics of the applied transducer. This may be taken into account in daily clinical routine by multiplying the theoretical resolution with an empirically obtained factor of 3 to 5, depending on the system used. Consequently, gallstones with a diameter of less than 3 mm, can be diagnosed reliably with conventional ultrasound systems, provided a 5 MHz transducer is applied.

Despite these basic considerations microlithiasis does not exhibit the characteristic sonographic pattern of gallstones, such as strong surface reflections and distal acoustic shadowing. In most cases, the acoustic shadow is lacking[4], and intraluminal echos, which represent microlithiasis, can be distinguished from polyps only by their mobility.

An even greater problem, raised in the cited paper, is the differentiation of stone compounds by imaging modalities in vivo. Although computed tomography is able to detect calcified gallstones, conventional radiography does not provide reliable data. In a series by Trotmann[5], 33% of radioopaque stones consisted mainly of cholesterol; Bell and colleagues[6] showed, that 12.5% of radiolucent stones contained more than 40% calcium. Computed tomography cannot discern pure cholesterol stones (see above).

The data concerning conventional B-mode ultrasound are conflicting. Purdom *et al.*[7] and our group[8] showed that the composition of gallstones had an influence on their B-mode image. However, calcium-bilirubinate could not be detected with satisfactory accuracy. Filly *et al.*[9] and Good *et al.*[10] could not find a correlation between the chemical stone composition and the reflection on the display. Therefore, a reliable differentiation of spatial distribution and stone compounds in microlithiasis with conventional imaging methods seems not to be possible at the present time. Wheather RF-signal processing[11] will solve this problem has to be shown in the future.

### *References*

1. Ochsner, S.F. (1977). Performance and reliability of cholecystography. *South. Med. J.*, **63**, 1268–1272
2. Sarva, R.P., Farivar, S., Fromm, H., Poller, W. (1981). Study of the sensitivity and specificity of computerized tomography in the detection of calcified gallstones which appear radiolucent by conventional roentgenography. *Gastrointest. Radiol.*, **6**, 165–167
3. Sutilov, V. Physik des Ultraschalls Springer, Heidelberg – New York 1984
4. Grossman, M. (1978). Cholelithiasis and acoustic shadowing. *J. Clin. Ultrasound,* **6**, 143–214
5. Trotmann, B., Petrella, E., Soloway, R.D., Sanchez, H.M., Morris, T.A., Miller, W.T. (1975). Evaluation of radiographic lucency or opaqueness of gallstones as a means of identifying cholesterol or pigment stones. *Gastroenterology,* **68**, 1563–1566
6. Bell, G.D., Dowling, R.H., Whitney, D., Sutor, D.J. (1975). The value of radiology in predicting gallstone type when selecting patients for medical treatment. *Gut,* **16**, 359–364
7. Purdom, R., Stephen, R.T., Kerelakes, J.G., Spitz, H.G., Goldenberg, N.J., Krugh, K.B. (1980). Ultrasonic properties of biliary calculi. *Radiology,* **136**, 729–732
8. Swobodnik, W., Ortmann, H., Wechsler, J.G., Techentrupp, K., Klüppelberg, U., Wenzel, H., Ditschuneit, H. (1986). Sonographie von Gallenblasensteinen: Möglichkeiten und Grenzen der Auswahl konservativ lysierbarer Steinträger. *Ultraschall,* **7**, 117–122
9. Filly, R.A., Moss, A., Way, L. (1979). *In vitro* investigation of gallstone shadowing with ultrasound tomography. *J. Clin. Ultrasound,* **7**, 255–261

10. Good, L.I., Edell, S.L., Soloway, R.D., Trotmann, B.W., Mulhern, C., Arger, R. (1979). Ultrasonic properties of gallstones. Effect of stone size an composition. *Gastroenterology*, **68**, 258–263
11. Swobodnik, W., Wechsler, J.G., Beuter, K., Wöhrle, U., Klüppelberg, U., Ditschuneit, H. HF-Signalanalyse von Gallenblasensteinen zur Bestimmung ihrer Zusammensetzung. in: R. Otto, P. Schnaars (Hrsg.): Ultraschalldiagnostik 85, Thieme, Stuttgart-New York (1986), 214

W. Swobodnik,
Department of Gastroenterology
University of Ulm
Postfach 3880
D-7900 Ulm, F.R.G.

